# Association of apolipoprotein A1 -75 G/A polymorphism with susceptibility to the development of acute lung injury after cardiopulmonary bypass surgery

**DOI:** 10.1186/1476-511X-12-172

**Published:** 2013-11-11

**Authors:** Jie Tu, Bingdong Zhang, Yanhua Chen, Beiwei Liang, Dongke Liang, Guofeng Liu, Fang He

**Affiliations:** 1 Institute of cardiovascular Diseases, The First Affiliated Hospital of Guangxi, Medical University, 6 ShuangYong Road, Nanning, Guangxi 530021, China

**Keywords:** Apolipoprotein A1, Acute lung injury, Cardiopulmonary bypass, Gene polymorphism

## Abstract

**Introduction:**

Apolipoprotein A1 (apoA1) is the major apoprotein constituent of high density lipoprotein (HDL) which exerts innate protective effects in systemic inflammation. However, its role in the acute lung injury (ALI) or acute respiratory distress syndrome (ARDS) has not been well studied. The objective of this study was to investigate the potential association between *APOA1* -75 G/A polymorphism and the development of ALI after cardiopulmonary bypass (CPB) surgery.

**Materials and methods:**

A hospital-based case–control study was conducted in patients with ALI (n = 300), patients without ALI (n = 300) and healthy controls (n = 300). Polymerase chain reaction restriction fragment length polymorphism (PCR-RFLP) assay was applied to assess the *APOA1* -75 G/A genotypes.

**Results:**

Patients with ALI had a significantly higher frequency of *APOA1* -75 AA genotype [odds ratio (OR) =1.75, 95% confidence interval (CI) = 1.04, 2.92; *P* = 0.03] than patients without ALI. *APOA1* -75 AA genotype (OR =3.47, 95% CI = 1.60, 7.52; *P =* 0.002) and A allele (OR =1.92, 95% CI = 1.24, 2.96; *P =* 0.003) were the significant independent prognostic factors for the 30-day survival rate of patients with ALI after CPB surgery.

**Conclusion:**

Our study suggested that *APOA1* -75 AA genotype was associated with a higher ALI risk after CPB surgery. Patients with the *APOA1* -75 AA genotype and A allele had higher 30-day mortality of ALI after CPB surgery. Additional studies are needed to confirm this finding.

## Introduction

Acute lung injury (ALI) is a common complication after cardiopulmonary bypass (CPB) surgery. ALI remains the main cause of mortality after CPB surgery [1]. The main causes of ALI after CPB surgery have been identified, including ischemia reperfusion injury, endotoxemia, primary pulmonary disease, surgical injury, and the systemic inflammatory reaction initiated by the contact of the blood leukocytes with the artificial surface of the bypass circuit [2-5]. Improved understanding of disease heterogeneity through use of evolving biologic, genomic, and genetic approaches should provide major new insights into pathogenesis of ALI [6].

Apolipoprotein A1 (apoA1) is the major apoprotein constituent of high density lipoprotein (HDL) which exerts innate protective effects in systemic inflammation [7]. However, its role in the acute lung injury (ALI) or acute respiratory distress syndrome (ARDS) has not been well studied. One variant resides in the *APOA1* gene, which involves a guanine to adenine transition 75 base pairs (bp) upstream from the start of transcription (G–75A) and destroys a site for the *Msp*I restriction enzyme. A strong association was found between the G to A substitution at -75 bp with serum HDL and apoA1 levels [8]. The objective of this study was to investigate the potential association between *APOA1* -75 G/A polymorphism and the development of ALI after cardiopulmonary bypass (CPB) surgery.

## Materials and methods

### Study population

From January 2008 to January 2013, a hospital-based case–control study was conducted in patients with ALI (n = 300), patients without ALI (n = 300) after CPB surgery and healthy controls (n = 300) in the Institute of cardiovascular Diseases of the First Affiliated Hospital of Guangxi Medical University. All subjects were collected from the same geographic region. Surgery type included valvular surgery, coronary artery bypass graft (CABG) and aortic surgery. The healthy control subjects were matched with the patients for age and sex. Healthy control subjects were recruited from the First Affiliated Hospital of Guangxi Medical University where they were attending a clinic for routine examination. Patients who met diagnostic criteria of acute lung injury at 24 hours after surgery were allocated to ALI group; those without ALI were allocated to without ALI group. ALI was defined as PaO_2_/FiO_2_ < 300 mm Hg; and bilateral pulmonary infiltrates on chest radiograph in the absence of acute pulmonary edema after left cardiac failure or other nonlung pathology 24 hours after surgery, and a left atrial pressure lower than 18 mm Hg [9]. Patients were excluded if they met the following criteria: immunodeficiency, autoimmune disease, or immunosuppressive therapy; tuberculosis, chronic obstructive pulmonary disease (COPD), or other chronic pulmonary diseases; liver dysfunction or chronic renal disease; bleeding disorders; anemia; postoperative pericardial tamponade requiring reoperation; postoperative low cardiac output syndrome, or acute pulmonary edema after left cardiac failure. The Ethical Committee of the First Affiliated Hospital of Guangxi Medical University approved the study protocols, and all participants gave written informed consent according to the Declaration of Helsinki.

### DNA extraction and genotyping

The commercially available Qiagen kit (QIAGEN Inc., Valencia, CA, USA) was used to extract DNA from peripheral blood leukocytes. The *APOA1* -75 G/A genotypes were analyzed by polymerase chain reaction-restriction fragment length polymorphism (PCR-RFLP) assay. Based on the GenBank reference sequence, the PCR primers were as follows: forward-5′- AGGGACAGAGCTGATCC-TTGAACTCTTAG -3′ and reverse-5′- TTAGGGGACACCTACCCGTACAGGAAGAGCA -3′. DNA was denaturanted at 94°C for 5 minutes, followed by 35 cycles of denaturation at 94°C for 1 minute, annealing at 60°C for 0.5 minute, and extension at 72°C for 0.5 minutes, with a final extension step of 5 minutes at 72°C. A total volume of 10 μl containing 20 U *Msp*I was added directly to the PCR product and digested at 37°C overnight. After electrophoresis, the digested products were visualized on a 9% polyacrylamide gel with ethidium bromide staining.

### Statistical analysis

We used Statistical Analysis System software (Version 8; SAS Institute Inc., Cary, NC, USA) to perform all of the statistical analyses. The *x*^2^ test was used to test for deviation of genotype frequencies from Hardy-Weinberg equilibrium and to compare the genotype distributions among patients with ALI, patients without ALI and healthy controls. We applied multivariate logistic regression to calculate crude and adjusted odds ratios (OR) and 95% confidence intervals (CI) for the association between the genotypes and the development of ALI after CPB surgery. A *P*-value was considered significant at a level of < 0.05.

## Results

Clinical data of patients with or without ALI after CPB surgery were listed in Table 1. Univariate study was performed to identify that age (*P =* 0.005), NYHA (*P =* 0.02), obesity (*P =* 0.03), peripheral vascular disease (*P =* 0.03), duration of operation (*P =* 0.003), and duration of CPB (*P =* 0.04) were associated with ALI after CPB surgery (Table 1). In addition, no significant differences were found between the patients with or without ALI after CPB surgery in gender, ASA, LVEF, previous cardiac surgery, hypertension, diabetes mellitus, transfusion, duration of cross-clamp, and surgery type (Table 1).

**Table 1:** Clinical data of patients with or without ALI after CPB surgery

**Variable**	**ALI**	**Without ALI**	***P***
Total no.	300	300	
Age (years)	59.1 ± 10.6	56.8 ± 9.4	0.005
Gender (Male/Female)	205/95	201/99	0.73
ASA	2.77 ± 0.71	2.67 ± 0.65	0.07
NYHA	2.69 ± 0.67	2.57 ± 0.55	0.02
LVEF (%)	59.68 ± 7.94	60.68 ± 8.04	0.13
Previous cardiac surgery (%)	45 (15.0)	30 (10.0)	0.07
Hypertension (%)	75 (25.0)	70 (23.3)	0.63
Diabetes mellitus (%)	26 (8.7)	24 (8.0)	0.77
Obesity (%)	70 (23.3)	48 (16.0)	0.03
Peripheral vascular disease (%)	43 (14.3)	26 (8.7)	0.03
Transfusion (mL)	694.3 ± 103.7	687.3 ± 99.8	0.40
Duration of operation (min)	231.7 ± 90.1	210.5 ± 82.3	0.003
Duration of CPB (min)	106.7 ± 43.7	99.5 ± 41.5	0.04
Duration of cross-clamp (min)	62.1 ± 32.4	60.7 ± 31.9	0.59
Surgery			
Valvular surgery (%)	176 (58.7)	183 (61.0)	0.56
CABG surgery (%)	99 (33.0)	102 (34.0)	0.80
Aortic surgery (%)	25 (8.3)	15 (5.0)	0.11

*ALI*, Acute lung injury; *CPB*, Cardiopulmonary bypass; *ASA*, American Society of Anesthesiology; *NYHA*, New York Heart Association; *LVEF*, Left ventricular ejection fraction; *CABG*, Coronary artery bypass graft.

Patients with ALI had a significantly higher frequency of *APOA1* -75 AA genotype (OR =1.75, 95% CI = 1.04, 2.92; *P =* 0.03) than patients without ALI (Table 2). *APOA1* -75 AA genotype (OR =3.47, 95% CI = 1.60, 7.52; *P =* 0.002) and A allele (OR =1.92, 95% CI = 1.24, 2.96; *P =* 0.003) were the significant independent prognostic factors for the 30-day survival rate of patients with ALI after CPB surgery (Table 3).

**Table 2:** Genotype and allele frequencies of apolipoprotein A1 -75 G/A polymorphism among patients with or without ALI after CPB surgery and controls

	**ALI (n = 300)**	**Without ALI (n = 300)**	**Healthy controls (n = 300)**	**ALI vs without ALI**		**ALI vs healthy controls**		**Without ALI vs healthy controls**	
				**OR (95% CI)**	***P*** **value**	**OR (95% CI)**	***P*** **value**	**OR (95% CI)**	***P*** **value**
Genotype									
GG	132(44.0)	144(48.0)	138(46.0)	1.00(Reference)		1.00(Reference)		1.00(Reference)	
GA	120(40.0)	126(42.0)	129(43.0)	1.04(0.74,1.47)	0.83	0.97(0.69,1.37)	0.87	0.94(0.67,1.31)	0.70
AA	48(16.0)	30(10.0)	33(11.0)	1.75(1.04,2.92)	0.03	1.52(0.92,2.52)	0.10	0.87(0.50,1.51)	0.62
Allele									
G	384(64.0)	414(69.0)	405(67.5)	1.00(Reference)		1.00(Reference)		1.00(Reference)	
A	216(36.0)	186(31.0)	195(32.5)	1.25(0.99,1.59)	0.07	1.17(0.92,1.48)	0.20	0.93(0.73,1.19)	0.58

*ALI*, Acute lung injury; *CPB*, Cardiopulmonary bypass; *vs*, versus; *OR*, Odds ratio; *CI*, Confidence interval.

**Table 3:** Effect of apolipoprotein A1 -75 G/A polymorphism on the 30-day survival rate of patients with ALI after CPB surgery

	**Non-survivors (n = 50)**	**Survivors (n = 250)**	**OR (95% CI)**	***P*** **value**
Genotype				
GG	18(36.0)	114(45.6)	1.00(Reference)	
GA	15(30.0)	105(42.0)	0.91(0.43,1.89)	0.79
AA	17(34.0)	31(12.4)	3.47(1.60,7.52)	0.002
Allele				
G	51(51.0)	333(66.6)	1.00(Reference)	
A	49(49.0)	167(33.4)	1.92(1.24,2.96)	0.003

*ALI*, Acute lung injury; *CPB*, Cardiopulmonary bypass; *OR*, Odds ratio; *CI*, Confidence interval.

## Discussion

Some studies have been performed to find an association of genetic polymorphism and ALI [10]. A prospective case–control study found that -607C/C genotype in IL-18 gene played a pivotal role in the development of ALI after CPB surgery in Chinese Han population [11]. Another case–control study found that the IL-6 -572 polymorphism was associated with ALI after CPB surgery [12]. Several studies have suggested that pre-B-cell colony-enhancing factor (PBEF) gene polymorphisms were associated with susceptibility to and prognosis of ALI [13,14]. The plasminogen activator inhibitor-1 (PAI-1) 4G allele was associated with worse outcome in ALI/ARDS [15]. A prospective cohort demonstrated that the AC genotype at position -1221 in the NQO1 gene caused decreased transcription and was associated with a lower incidence of ALI following major trauma [16]. In a nested case–control study, patients with the NRF2 -617 A allele had a significantly higher risk for developing ALI after major trauma [17]. A case–control study found that myosin light chain kinase (MYLK) genetic variants were associated with increased risk of sepsis-associated ALI [18].

The *APOA1* -75 G/A polymorphism has recently been linked to many other diseases. A comparative study found that carrying the *APOA1* -75 A allele could confer a higher risk of hyperlipidemia in obese children [19]. A prospective case–control study found that the *APOA1* -75 G/A polymorphism influenced cholesterol metabolism [20]. A study in healthy Tamilian volunteers of south India found that the *APOA1* -75 G/A polymorphism was significantly associated with HDL-C levels [21]. A study found the *APOA1* -75 G/A polymorphism was significantly associated with plasma triglyceride levels in men with coronary artery disease from the REGRESS study [22]. A case–control study found the *APOA1* -75G/A promoter polymorphism was associated with variations in serum triglyceride concentrations in hypercholesterolemic individuals [23]. A case–control study suggested that a positive association was found between the *APOA1* -75 A allele carriers and breast cancer risk [24]. A pilot study in a north Indian population suggested that the *APOA1* -75 G allele might be susceptibility alleles for myocardial infarction [25]. A case–control study found an association of the *APOA1* -75G/A promoter polymorphism with cognitive performance in multiple sclerosis [26]. A cohort study found that the *APOA1* -75 G allele showed significant association with hypertension [27]. A case–control study found the *APOA1* -75 G/A polymorphism was associated with gallstone disease [28]. A case–control study found the *APOA1* -75 A allele was associated with an increased risk for Alzheimer’s disease [29]. A case–control study found the *APOA1* -75 G/A polymorphism was significantly associated with lipid levels and coronary atherosclerosis disease [30].

Some limitations of this study should be noted. First of all, these results should be interpreted with caution because the population was only from China, which reduces the possibility of confounding from ethnicity, so it does not permit extrapolation of the results to other ethnic groups. Second, the sample size of this study is relatively small, which may not have enough statistical power to explore the real association. Third, this is a hospital based case control study, so the selection bias cannot be avoidable and the subjects may not be representative of the general population.

In conclusion, our study suggested that *APOA1* -75 AA genotype was associated with a higher ALI risk after CPB surgery. Patients with the *APOA1* -75 AA genotype and A allele had higher 30-day mortality of ALI after CPB surgery. Additional studies are needed to confirm this finding.

## Competing interest

The authors declare that they have no competing interests.

## Authors’ contributions

JT and BZ carried out the molecular genetic studies and drafted the manuscript. YC and BL carried out the genotyping. DL, GL participated in the design of the study and performed the statistical analysis. FH conceived of the study, and participated in its design and coordination and helped to draft the manuscript. All authors read and approved the final manuscript.

## Acknowledgement

This research received no specific grant from any funding agency in the public, commercial, or not-for-profit sectors.
